# Supplementary material for: Increased sperm production linked to competition in the maternal social environment
Source: R Soc Open Sci. 2020 Dec 16;7(12):201171. doi: 10.1098/rsos.201171 (PMC7813238; doi:10.1098/rsos.201171)
Supplement: Results of full models (those containing all random and fixed effects) analysing the effects of the maternal social environment on reproductive traits of adult male offspring. [file rsos201171supp1.docx]

**Supplementary material 1**: Results of full models (those containing all random and fixed effects) analysing the effects of the maternal social environment on reproductive traits of adult male offspring.

Table S1. Effect of maternal social environment on adult male offspring epididymides mass. The litter and block in which the male offspring were born were included as random effects in the model. The maternal social environment (treatment) and body weight were included as fixed effects. The variance and standard deviation (std dev) calculated as part of the model are provided for all random effects. The estimate, standard error (std error) and T-value calculated as part of the model are provided for all fixed effects.

| **Random Effects** | | | |
| --- | --- | --- | --- |
| **Group** | **Variance** | **Std dev** | |
| Litter | 0.000 | 0.000 | |
| Block | 0.000 | 0.000 | |
| Residual | 0.008 | 0.092 | |
| **Fixed Effects** | | | |
| **Effect** | **Estimate** | **Std error** | **T value** |
| Intercept | -2.006 | 0.794 | -2.526 |
| Treatment(low) | -0.084 | 0.038 | -2.200 |
| Log(Body weight) | 0.885 | 0.263 | 3.362 |

Table S2. Effect of maternal social environment on adult male offspring daily rate of sperm production. The litter and block in which the male offspring were born were included as random effects in the model. The maternal social environment (treatment) and testes mass were included as fixed effects. The variance and standard deviation (std dev) calculated as part of the model are provided for all random effects. The estimate, standard error (std error) and T-value calculated as part of the model are provided for all fixed effects.

| **Random Effects** | | | |
| --- | --- | --- | --- |
| **Group** | **Variance** | **Std dev** | |
| Litter | 0.000 | 0.000 | |
| Block | 1.273e-19 | 3.568e-10 | |
| Residual | 1.702e-02 | 1.305e-01 | |
| **Fixed Effects** | | | |
| **Effect** | **Estimate** | **Std error** | **T value** |
| Intercept | 18.037 | 0.388 | 46.525 |
| Treatment(low) | -0.149 | 0.0523 | -2.840 |
| Log(Testes mass) | 1.716 | 0.291 | 5.893 |

Table S3. Effect of maternal social environment on adult male offspring body mass. The litter and block in which the male offspring were born were included as random effects in the model. The maternal social environment (treatment) was included as a fixed effect. The variance and standard deviation (std dev) calculated as part of the model are provided for all random effects. The estimate, standard error (std error) and T-value calculated as part of the model are provided for all fixed effects.

| **Random Effects** | | | |
| --- | --- | --- | --- |
| **Group** | **Variance** | **Std dev** | |
| Litter | 0.000 | 0.000 | |
| Block | 0.001 | 0.024 | |
| Residual | 0.004 | 0.064 | |
| **Fixed Effects** | | | |
| **Effect** | **Estimate** | **Std error** | **T value** |
| Intercept | 3.002 | 0.027 | 110.554 |
| Treatment(low) | -0.025 | 0.027 | -0.942 |

Table S4. Effect of maternal social environment on adult male offspring testes mass. The litter and block in which the male offspring were born were included as random effects in the model. The maternal social environment (treatment) and body mass were included as fixed effects. The variance and standard deviation (std dev) calculated as part of the model are provided for all random effects. The estimate, standard error (std error) and T-value calculated as part of the model are provided for all fixed effects.

| **Random Effects** | | | |
| --- | --- | --- | --- |
| **Group** | **Variance** | **Std dev** | |
| Litter | 0.000 | 0.000 | |
| Block | 0.000 | 0.000 | |
| Residual | 0.005 | 0.070 | |
| **Fixed Effects** | | | |
| **Effect** | **Estimate** | **Std error** | **T value** |
| Intercept | -2.831 | 0.607 | -4.662 |
| Treatment(low) | 0.024 | 0.029 | 0.811 |
| Log(Body weight) | 0.721 | 0.201 | 3.584 |

Table S5. Effect of maternal social environment on adult male offspring seminal vesicle mass. Effect of maternal social environment on adult male offspring testes mass. The litter and block in which the male offspring were born were included as random effects in the model. The maternal social environment (treatment) and body mass were included as fixed effects. The variance and standard deviation (std dev) calculated as part of the model are provided for all random effects. The estimate, standard error (std error) and T-value calculated as part of the model are provided for all fixed effects.

| **Random Effects** | | | |
| --- | --- | --- | --- |
| **Group** | **Variance** | **Std dev** | |
| Litter | 0.283 | 0.168 | |
| Block | 0.000 | 0.000 | |
| Residual | 0.109 | 0.330 | |
| **Fixed Effects** | | | |
| **Effect** | **Estimate** | **Std error** | **T value** |
| Intercept | -5.623 | 3.077 | -1.827 |
| Treatment(low) | 0.168 | 0.168 | -1.001 |
| Log(Body weight) | 1.259 | 1.021 | 1.233 |
